# Peer Teaching in an Interprofessional Education Activity Focused on Professional Skills Development

**DOI:** 10.3390/pharmacy9020112

**Published:** 2021-06-16

**Authors:** Tracey DelNero, Deepti Vyas

**Affiliations:** 1 School of Health Sciences, University of the Pacific, Sacramento, CA 95817, USA; tdelnero@pacific.edu; 2 School of Pharmacy, University of the Pacific, Stockton, CA 95211, USA

**Keywords:** peer teaching, cross-teaching, interprofessional education, pharmacy student, physician assistant student

## Abstract

The purpose of this study was to assess the impact of a peer-taught interprofessional education (IPE) activity on pharmacy and physician assistant (PA) student self-assessed confidence and attitudes related to professional skills. First-year pharmacy (*n* = 210) and PA (*n* = 45) students participated in a two-hour IPE activity. Forty-five teams consisting of one PA and at least four pharmacy students completed three peer-teaching stations focused on diabetes device education, pulmonary device teaching/case workup, and physical assessment skills. Students completed a pre- and post-activity confidence survey and a post-activity attitudes survey. For pharmacy students, highest confidence gains were noted on the items related to performing a physical exam. For PA students, largest gains were noted on the items related to insulin delivery systems. Eighty-three percent of students either agreed or strongly agreed with the statement “I learned things during this IPE activity that I will implement in clinical practice.” Seventy six percent of students felt that the physical assessment station was “beneficial or very beneficial” to their learning. A vast majority of students noted the IPE activity “somewhat or definitely” enhanced their communication with other health professionals and promoted a climate of mutual respect. In conclusion, peer teaching improved student attitudes and confidence.

## 1. Introduction

Pharmacists and physician assistants (PA) routinely collaborate through patient-centered communications or as members of interprofessional teams. It is imperative that programs offer opportunities for students in these professions to collaborate and learn from each other. The pharmacy and PA accreditation agencies both value interprofessional education (IPE) and require inclusion of IPE in the didactic and experiential curriculum [1,2]. IPE promotes students’ exchange of knowledge, promotes mutual role comprehension and respect, and reduces isolationism [3]. Interactive IPE activities enhance learner knowledge, individual and team communication skills, and facilitate team dynamics [3]. However, provision of IPE is fraught with barriers such as limited faculty/instructor numbers, scheduling issues, and lack of time in individual health professions curricula [4,5,6,7,8]. Therefore, use of tools such as peer teaching which reinforce curricular concepts with fewer faculty resources can be useful for educators looking to address specific barriers to IPE provision [4,5,6,7,8]. Peer teaching is defined as active teaching by students who are at the same/similar academic level as those they are teaching [4,5,6,7,8]. Peer teaching can be supported by the cognitive and social congruence theory [9]. In peer teaching, cognitive congruence implies that the peer-teachers share the same/similar knowledge base and language as those they are teaching. Social congruence refers to the peer-teacher and student sharing similar social roles and norms [9]. Cognitive and social congruence suggest that learning through the same knowledge framework within similar social roles can allow students to overcome their learning deficiencies and reinforce existing knowledge [9]. In medical education, Burgess suggested that peer teaching can be beneficial in the development of professional identity including recognition of future roles as medical practitioners [10]. In pharmacy education, Sadowski and colleagues highlighted a peer-teaching program with physical therapy students providing ambulatory device training to pharmacy students. Significant improvement was noted on pre/post-activity scores as well as student satisfaction with the IPE activity [4]. Similarly, Lipton and colleagues found that training provided by pharmacy students improved medical and nursing student knowledge of Medicare Part D [5]. In peer-led seminars involving medical and pharmacy students, Lehrer and colleagues, reported improved interprofessional attitudes as measured by the Interdisciplinary Education Perception Scale [6]. In a pharmacy student-led peer-taught pharmacology course, Hsia reported positive feedback and improvement on the student perceptions of physician-pharmacist interprofessional clinical education (SPICE) survey [7]. However, there is a dearth of studies describing the impact of interprofessional peer teaching in professional skills acquisition and reinforcement. Professional skills such as performing a physical exam or providing appropriate device education are paramount in preventing patient harm [11,12,13]. Prescription errors and deficiencies in patient education surrounding appropriate insulin administration can lead to poor disease management and patient harm [11]. Insulin errors can occur from prescribing to administration, including device selection, prescribing, dispensing, storage, and administration. Various factors may play a role in erroneous administration with a lack of patient education being one of the most common reasons [11]. As insulin is paramount to diabetic management, inhaled medication therapy is fundamental to obstructive pulmonary disease management. Inhaled pulmonary medications are administered through aerosolization using either a meter dose inhaler (MDI) or dry powder inhaler (DPI) [12]. Neither medication delivery system facilitates easy use. The MDI requires a precise correlation of actuation and appropriate timing, rate, and inhalation force [12]. DPIs eliminate the timing correlation but require a fast rate and increased inhalation force to propel the powder into the lower airway rather than remaining in the oral cavity [12]. We hypothesize that interprofessional peer teaching to reinforce these difficult concepts could provide a multifaceted approach to training students on these important professional skills. The purpose of this study is to describe the impact of a peer-taught professional skills-based IPE activity on pharmacy and PA student confidence and attitudes.

## 2. Materials and Methods

The institutional review board at the University of the Pacific approved this study. Pharmacy and PA students participated in a two-hour peer-teaching IPE activity in the summer semester of their first year. The pharmacy program comprises of a six-semester didactic program starting in the Fall semester and the PA program consists of a 3.5 semester didactic curriculum starting in the Spring semester. An IPE page within the university’s learning management system (LMS) facilitated communication and team formation. Random teams consisting of one PA student and four to five pharmacy students were created by course faculty. Prior to this peer-teaching IPE activity, these same interprofessional teams had already met once in the Spring semester for an activity focused on understanding the professions’ scope of practice and team patient-care decision-making activities. The current activity focused on peer cross-teaching of select professional skills. Each program highlighted professional skills which needed reinforcement in their core-curriculum. The pharmacy program identified physical assessment skills while the PA program identified device and patient education related to diabetes and pulmonary disease as areas requiring reinforcement. Both sets of students had already received training on each of these areas in their respective curricula. The pharmacy students had received this training one month before the IPE activity while the PA students received physical assessment training in the Spring semester and instruction on pulmonary and diabetes devices one month before the IPE in the Summer semester. 

Students were asked to complete a knowledge and skills confidence survey ten days before the IPE activity. The survey was administered through the LMS. The pre-activity survey consisted of twelve confidence questions rated on a 5-point Likert scale (1 = not confident to 5 = very confident). The pre-activity survey was created by course faculty to measure confidence on each of the items taught in the IPE activity.

**Figure 1:**
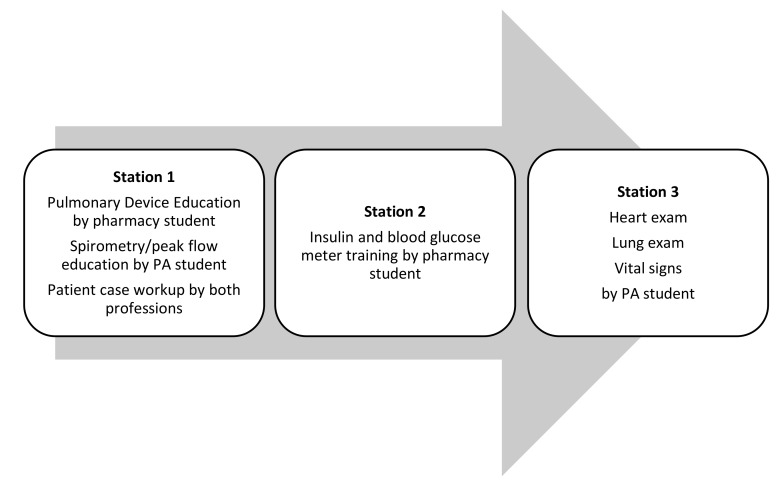
Peer-Teaching Stations. PA = physician assistant.

A week before the IPE activity, instructors informed teams about the topics that they would be teaching, learning outcomes of each station, and critical components on which to focus. The three stations were (1) physical assessment, (2) inhaler and other pulmonary device teaching and case analysis, and (3) insulin and other diabetes device education. Course faculty members consisting of one PA and one pharmacy instructor developed goals, teaching and learner objectives, key instructional topics, and instructional activities for all three station materials and survey questionnaires. Teams rotated between the three stations every 35 min. Stations were completed in a standard order as determined by the first assigned station (Figure 1). Each station consisted of instructional content provided by the peer-teacher and a knowledge application activity, either skills practice or simulated patient education by all team members. PA students provided clinical background and instruction on appropriate techniques for vital sign collection, cardiovascular, and pulmonary patient examination, while pharmacy students provided training surrounding administration of and patient education for insulin. Pharmacy students also provided education on appropriate administration of inhaled pulmonary medications and pulmonary devices while the PA students provided education on the interpretation of spirometry and peak-flow diagnostic studies. An additional educational component consisted of patient-centered data interpretation and knowledge application through a short patient case analysis, requiring the expertise of both professions.

After the IPE activity, students had ten days to complete a post-activity confidence and attitudes survey. As with the pre-activity survey, students received an announcement, completed it through the LMS. The post-activity confidence survey consisted of the twelve items from the pre-activity survey confidence survey using the same evaluation scale. The attitudes survey consisted of four questions regarding the impact of the IPE activity using a 5-point Likert scale (1 = definitely worsened to 5 = definitely enhanced). Three more questions measured the benefit of each station towards learning and future clinical practice rated on a 5-point scale (1 = not beneficial to 5 = very beneficial). Finally, two questions measured student agreement with the statements “I learned things during this IPE event that I will implement in clinical practice” and “This interprofessional (IPE) objective structured learning experience (OSLE) was useful to my learning” on a 5-point Likert scale (1 = strongly disagree to 5 = strongly agree).

Student data were exported from the LMS for statistical analysis. All surveys contained student identifiers to allow for pre/post-activity analysis, when applicable. A paired Student *t*-test was used to evaluate any changes in the students’ pre/post-activity confidence ratings. Descriptive statistics including means, standard deviation, and percentage were used to describe the attitudes survey. 

### Resources Required

This IPE activity required fifteen sets of physical exam tables, MDI or DPI, peak flow meters, insulin vials/syringes, blood glucose monitoring supplies, blood pressure monitors, and paper cases. Three station monitors (instructors from the pharmacy and PA program) answered student questions and replenished supplies as needed. Three additional monitors (graduate pharmacy teaching assistants) kept time and ensured appropriate rotation between stations.

## 3. Results

Two hundred and fifty-five first-year health professions students (210 pharmacy and 45 PA students) participated in this IPE activity. The response rate (RR) differed across both groups and is provided in brackets. On the attitudes survey, eighty-three percent (*N* = 211, mean = 4.2/5, RR = 100%) of students either agreed or strongly agreed with the statement “I learned techniques during this IPE activity that I will implement in clinical practice”. Seventy-six percent (*N* = 193, mean = 4.08/5, RR = 100%) of students either agreed or strongly agreed with the statement “This interprofessional objective structured learning experience (OSLE) was useful to my learning.” On the evaluation of each learning station, 76% of students felt the physical assessment station was “beneficial or very beneficial” to their learning (*N* = 193, mean = 4.1/5, RR = 100%). Seventy percent rated the pulmonary station as “beneficial or very beneficial” to their learning (*N* = 178, mean 3.9/5, RR = 100%). Seventy-one percent rated the insulin station as “beneficial or very beneficial” to their learning (N = 181, mean = 3.9/5, RR = 100%). A vast majority of students noted the IPE activity “somewhat or definitely” enhanced their communication with other health professionals (*N* = 242; 95%, mean = 4.46/5, RR = 100%), promoted a climate of mutual respect (*N* = 242; 95%, mean = 4.54/5, RR = 100%), and their ability to apply the team’s knowledge to patient care (*N* = 244; 96%, mean = 4.49/5). Ninety-four percent (*N* = 239, mean = 4.43/5, RR = 100%) of students reported that the IPE activity enhanced their ability to perform effectively in a health care team. Table 1 reports the mean student attitudes scores on the four questions outlining the impact of the IPE activity.

For all students, pre-activity confidence data showed lowest mean scores (mean 2.53) on the item “performing a heart exam on a patient” and highest scores (mean 3.87) on the item “Educating a patient on the appropriate administration of various inhaled medication delivery systems.” On the post-activity confidence data, lowest confidence (mean 3.43) was noted on the item “performing a heart exam on a patient” and the highest scores (mean 4.4) on the item “Educating a patient on the use of a glucose meter to test blood glucose.” Table 2 shows mean student confidence by profession along with probability analysis (RR = 98%). 

## 4. Discussion

Evidence suggests that peer teaching is an effective mechanism for improving knowledge and skills [4,5,6,7,8]. This IPE activity leveraged areas of curriculum strength to allow students to teach their peers in an interprofessional setting. The results demonstrate improved confidence in previously taught skills and enhanced interprofessional attitudes Student attitudes toward the activity’s benefit to their learning and future clinical practice exceeded expectations. The overall perceived benefit of the individual stations varied from 70 to 76%, with mean scores above average. The physical assessment station noted the most significant perceived benefit from all students. Eighty-three percent of the students noted clinical knowledge acquisition significant enough to impact future patient care. The potential for the broader impact of this IPE activity is the significant number of students reporting improved communication, enhanced climate of mutual respect, and even higher percentage reporting the heightened ability to effectively function in a health care team with mean scores nearing or exceeding 4.4 on a 5-point scale.

As demonstrated by the data reported in Table 2, the average scores increased on the post-activity survey from a half to nearly a whole point on a 5-point scale, except for confidence in “taking a manual blood pressure.” Pharmacy student confidence improved significantly on all but one item (measuring respiratory rate). These results demonstrate the educational value of peer teaching based on expertise. However, concerning are the lower overall pharmacy student scores on the items related to physical assessment skills and interpreting spirometry results. This is an area that requires more emphasis in the pharmacy curriculum.

A confidence data analysis differentiated by profession revealed statistically significant results. A probability analysis revealed statistically significant improvement (*p*-value < 0.01) for nearly all data points for both professions. The average scores increased regardless of whether the profession was the teacher or recipient of the educational content, supporting the idea that teaching others strengthens one’s own confidence. Pharmacy students average score increased by more than half a point on the 5-point scale for each of the subjects instructed by them. Similarly, PA students’ confidence increased from 0.3 to 0.8 points on most of the topics taught by them. The PA student’s confidence for “checking a patient’s heart rate and rhythm” registered a 0.2-point increase, which was not statistically significant despite being an increase. Post-activity survey data of PA student confidence surrounding insulin delivery systems and patient education regarding proper administration revealed a significant increase on the 5-point scale.

This study reinforced the utility of peer teaching on key topics which have been identified in the literature as problematic areas [11,12,13]. These studies stress the importance of training students fully on these key topics. This current peer teaching IPE activity provides interesting data on training students on device education and patient assessment. However, this study has some limitations. This study did not utilize a validated IPE survey which would have provided vital information regarding the impact of this IPE activity on interprofessional attitudes. In addition, evaluation of the accuracy of the information provided by the peer would have provided another layer regarding the utility of peer teaching. This will be the focus of future research. In addition, due to the discrepancy in pharmacy to PA student ratios, there was just one PA to 4–5 pharmacy students. This means that the team was relying on the expertise of just one PA student per team, which could be problematic. However, since both professions had already received training on each station in their respective curricula, instructors felt that grossly inaccurate information would be corrected by the rest of the team. Finally, long-term retention of the skills reinforced during the IPE activity was not measured. This limits the utility of this study. Use of an objective structured clinical exam (OSCE) would provide some data on actual gains in skills versus student perceived confidence. This is an area of future research. Future plans for the IPE include observers who would follow each team and provide an evaluation of the quality of peer teaching. In addition, an OSCE administered at the end of the activity will be added to the IPE event in order to ensure student accountability and provide evidence of skills development.

## 5. Conclusions

The educational benefits of this peer-teaching IPE activity on student confidence and attitudes are noteworthy. Student attitudes toward interprofessional health care teams and their ability to function within the team strengthened. Student confidence was enhanced through educating their peers and receiving education by those same peers. Future research should evaluate long-term retention of these gains using an OSCE or similar assessment.

## Figures and Tables

**Table 1:** Impact of the Interprofessional Education Peer-Teaching Stations on Student Attitudes (*N* = 255, RR = 100%).

Statement: Rate the Impact of the IPE Activities on Your Ability to: ^1^	Mean (SD)
Participate in building a patient care team and perform effectively in various roles on the team.	4.43 (0.6)
Effectively communicate with other health professionals to support a team approach to patient care.	4.45 (0.59)
Use the knowledge of one’s own role and those of other professions in providing patient care.	4.49 (0.57)
Work with individuals of other professions to maintain a climate of mutual respect and shared values.	4.54 (0.59)

^1^ Survey based on a Likert scale of 1–5, with 1 = did not enhance and 5 = definitely enhanced.

**Table 2:** Student Confidence Pre/Post-Peer-Teaching Interprofessional Education Activity.

Statement: What Is Your Current Confidence Level Doing the Following?	Pre-Activity (Pharmacy) Mean (SD) *N* = 207	Post-Activity (Pharmacy) Mean (SD) *N* = 207	Gains (Pharmacy)	Pre-Activity (PA) Mean (SD) *N* = 43	Post-Activity (PA) Mean (SD) *N* = 43	Gains (PA)
Differentiating the indications of various inhaled medication delivery systems	3.7 (0.78)	4.3 (0.73) *	0.6	3.2 (0.8)	3.7 (0.87) *	0.5
Identifying comorbidities influencing the use of various inhaled medication delivery systems	3.1 (0.77)	3.9 (0.79) *	0.8	3.3 (0.72)	3.7 (0.84) *	0.4
Educating a patient on the appropriate administration of various inhaled medication delivery systems	3.9 (0.8)	4.5 (0.64) *	0.6	3.5 (0.9)	4.0 (0.79) *	0.5
Differentiating the indications and use of various insulin delivery systems	3.4 (0.85)	4.2 (0.73) *	0.8	1.7 (1.04)	3.4 (0.89) *	1.7
Educating a patient on the appropriate administration and storage of various insulin delivery systems	3.6 (0.84)	4.3 (0.72) *	0.7	1.8 (1.21)	3.6 (0.84) *	1.8
Educating a patient on the use of a glucose meter to test blood glucose	3.9 (0.89)	4.5 (0.66) *	0.6	2.8 (1.32)	4.1 (0.82) *	1.3
Taking a manual blood pressure	3.7 (0.95)	4.1 (0.81) *	0.4	4.5 (0.59)	4.8 (0.47) *	0.3
Checking a patient’s heart rate and rhythm	3.3 (0.94)	3.9 (0.87) *	0.6	4.7 (0.49)	4.9 (0.35)	0.2
Assessing a patient’s respiratory rate	3.2 (0.93)	3.8 (0.90)	0.6	4.5 (0.66)	4.8 (0.37) *	0.3
Performing a heart exam on a patient	2.3 (0.92)	3.2 (1.03) *	0.9	3.5 (0.73)	4.3 (0.76) *	0.8
Performing a lung exam on a patient	2.3 (0.90)	3.2 (1.04) *	0.9	3.9 (0.73)	4.4 (0.62) *	0.5
Interpreting spirometry results	2.6 (0.94)	2.7 (0.93) *	0.1	3.6 (0.69)	3.9 (0.78) *	0.3

Student confidence on a 5-point Likert scale, with 1 = not at all confident and 5 = very confident. * Indicates *p* value < 0.01.

## Data Availability

Raw data is available here: https://docs.google.com/spreadsheets/d/1U893_CmDBEVF3tieAjdbNqmaap8VVXclmlXOZjz_Evs/edit?usp=sharing (accessed on 15 June 2021).

## References

[1] Accreditation Council for Pharmacy Education Accreditation Standards and Key Elements for the Professional Program in Pharmacy Leading to the Doctor of Pharmacy Degree. https://www.acpe-accredit.org/pharmd-program-accreditation/.

[2] Accreditation Review Commission on Education for the Physician Assistant, Inc. (2021). Accreditation Manual—Accreditation Standards for Physician Assistant Education, 5th Edition. http://www.arc-pa.org/wp-content/uploads/2021/03/Standards-5th-Ed-March-2021.pdf.

[3] Interprofessional Education Collaborative (2019). Core Competencies for Interprofessional Collaborative Practice. https://nebula.wsimg.com/2f68a39520b03336b41038c370497473?AccessKeyId=DC06780E69ED19E2B3A5&disposition=0&alloworigin=1.

[4] Sadowski C.A., Li J.C., Pasay D., Jones C.A. (2015). Interprofessional Peer Teaching of Pharmacy and Physical Therapy Students. Am. J. Pharm. Educ..

[5] Lipton H.L., Lai C.J., Cutler T.W., Smith A.R., Stebbins M.R. (2010). Peer-to-peer interprofessional health policy education for Medicare part D. Am. J. Pharm. Educ..

[6] Lehrer M.D., Murray S., Benzar R., Stormont R., Lightfoot M., Hafertepe M., Welch G., Peters N., Maio A. (2015). Peer-led problem-based learning in interprofessional education of health professions students. Med. Educ. Online.

[7] Hsia S., Tran D.N., Beechinor R., Gahbauer A., Fitzsimmons A., Brock T. (2020). Interprofessional peer teaching: The value of a pharmacy student-led pharmacology course for physical therapy students. Curr. Pharm. Teach. Learn..

[8] Burgess A., Roberts C., van Diggele C., Mellis C. (2017). Peer teacher training (PTT) program for health professional students: Interprofessional and flipped learning. BMC Med. Educ..

[9] Loda T., Erschens R., Loenneker H., Keifenheim K.E., Nikendei C., Junne F., Zipfel S., Herrmann-Werner A. (2019). Cognitive and social congruence in peer-assisted learning—A scoping review. PLoS ONE.

[10] Burgess A., Nestel D. (2014). Facilitating the development of professional identity through peer assisted learning in medical education. Adv. Med. Educ. Pract..

[11] Truong T.H., Nguyen T.T., Armor B.L., Farley J.R. (2017). Errors in the Administration Technique of Insulin Pen Devices: A Result of Insufficient Education. Diabetes Ther..

[12] Usmani O.S., Lavorini F., Marshall J., Dunlop W.C.N., Heron L., Farrington E., Dekhuijzen R. (2018). Critical inhaler errors in asthma and COPD: A systematic review of impact on health outcomes. Respir Res..

[13] Barry A.R., Egan G., Turgeon R.D., Leung M. (2019). Evaluation of Physical Assessment Education for Practising Pharmacists: A Cross-Sectional Survey. Can. J. Hosp. Pharm..

